# Reticular alignment: A progressive corner-cutting method for multiple sequence alignment

**DOI:** 10.1186/1471-2105-11-570

**Published:** 2010-11-23

**Authors:** Adrienn Szabó, Ádám Novák, István Miklós, Jotun Hein

**Affiliations:** 1 Computer and Automation Research Institute, Hungarian Academy of Sciences, Lágymányosi u. 11., 1111 Budapest, Hungary; 2 Eötvös Loránd University, Faculty of Informatics, Pázmány Péter sétány 1/c., 1117 Budapest, Hungary; 3 Department of Statistics, University of Oxford, 1 South Parks Road, OX1 3TG Oxford, UK; 4 Oxford Centre for Integrative Systems Biology, Department of Biochemistry, South Parks Road, OX1 3QU Oxford, UK; 5 Alfréd Rényi Institute of Mathematics, Hungarian Academy of Sciences, Reáltanoda u. 13-15., 1053 Budapest, Hungary

## Abstract

**Background:**

In this paper, we introduce a progressive corner cutting method called Reticular Alignment for multiple sequence alignment. Unlike previous corner-cutting methods, our approach does not define a compact part of the dynamic programming table. Instead, it defines a set of optimal and suboptimal alignments at each step during the progressive alignment. The set of alignments are represented with a network to store them and use them during the progressive alignment in an efficient way. The program contains a threshold parameter on which the size of the network depends. The larger the threshold parameter and thus the network, the deeper the search in the alignment space for better scored alignments.

**Results:**

We implemented the program in the Java programming language, and tested it on the BAliBASE database. Reticular Alignment can outperform ClustalW even if a very simple scoring scheme (BLOSUM62 and affine gap penalty) is implemented and merely the threshold value is increased. However, this set-up is not sufficient for outperforming other cutting-edge alignment methods. On the other hand, the reticular alignment search strategy together with sophisticated scoring schemes (for example, differentiating gap penalties for hydrophobic and hydrophylic amino acids) overcome FSA and in some accuracy measurement, even MAFFT. The program is available from http://phylogeny-cafe.elte.hu/RetAlign/

**Conclusions:**

Reticular alignment is an efficient search strategy for finding accurate multiple alignments. The highest accuracy achieved when this searching strategy is combined with sophisticated scoring schemes.

## Background

The multiple sequence alignment problem is still the Holy Grail of bioinformatics [1]. There are 517100 sequences in the UniProtKB/Swiss-Prot release of the 18th of May 2010 http://expasy.org/sprot/, while on the other hand, there are only 65802 known structures in the last PDB database relase of the 8th of June 2010 http://www.pdb.org/pdb/home/home.do. Therefore, the *in silico *prediction of protein structures is still demanding, and the majority of the protein structure prediction methods need accurate alignments. There are two major technical hurdles in the multiple sequence alignment problem. The first is the scoring problem: how to score the alignments such that the best scored alignment is the most accurate one. The second is the algorithmic problem: how to find the best scored alignment.

Significantly more effort has been put into the research for solving the second challenge. Although the number of possible alignments of two sequences grows exponentially with the length of the sequences, finding the best scoring alignment of two sequences is computationally feasible, since such an alignment can be found by iteratively comparing the prefixes of the two sequences [2]. The optimal alignment of longer prefixes can be calculated quickly from shorter prefixes, and hence, the algorithm needs only memory and running time that both are proportional to the product of the lengths of the sequences. This dynamic programming algorithm can be extended to many sequences [3], however, it becomes computationally infeasible, since analysing all possible combinations of prefixes requires *O*(*L^N^*) memory and running time. It has been proven that finding the best scoring multiple alignment under the sum-of-pairs scoring scheme is NP-hard [4], therefore it is very unlikely that any fast algorithm exists for the exact multiple sequence alignment problem.

The memory requirement and running time can be reduced by corner-cutting methods. Corner-cutting algorithms define a narrow strip in the dynamic programming table which contains the optimal alignment. Some methods use an *a priori *estimated upper limit for the score of the optimal alignment to define such a strip [5-7]. Hein *et al*. obtained a strip around the parsimony-based optimal alignment for HMM-based calculations [8]. The strip can also be defined *on the y *using the so-called diagonal extension method [9]. The corner-cutting method has been extended to multiple sequence alignment, too [10,11], with which the optimal alignment of 4-10, each 200-300 long sequences can be found in reasonable time [12]. However, even the size of the narrowest possible strip - which has a unit hypercube transverse section - grows exponentially with the number of sequences to be aligned, hence, this approach eventually becomes unfeasible for large number of sequences.

Above exact methods, approximation methods for the multiple sequence alignment problem are also widespread. The most commonly used approximation to multiple sequence alignment is the progressive alignment approach [13-17], which builds multiple sequence alignments bottom-up along a guide tree, through a series of pairwise alignments of two sequences (leaves of the guide tree), two alignments (inner nodes), or a sequence and an alignment. The guide tree is typically constructed from the pairwise distance matrix of the sequences that is computed using pairwise sequence alignments. These methods apply the "once a gap, always a gap" rule [14]: gaps inserted into an alignment at an inner node of the guide tree cannot be removed or modified further up in the guide tree. Although one can trust more in gaps introduced at the lower nodes of the guide tree, there is no guarantee that these gaps are correct, and a gap that has incorrectly been inserted into a subalignment based on local information cannot be corrected later on.

There have been successful attempts in other directions to reduce the computational time required to align sequences. MAFFT employs the Fast Fourier Transformation (FFT) technique to rapidly identify homologous regions by converting the amino acid sequence into a sequence of volume and polarity values [18]. The two basic optimisation heuristics (progressive and iterative alignment) have been substituted by more advanced iterative methods in the most recent version of the software where pairwise alignment information is incorporated into the objective function, thus making MAFFT one of the most accurate alignment tools available.

One artifact shared by all of the previously mentioned methods is that evolutionary events are scored using user-specified values (gap penalties and substitution matrices). The accuracy of the alignments largely depends on the selection of these parameter values. To overcome these difficulties the statistical alignment approach has been introduced where evolutionary models describe the type of events that transform the sequences and provide a means of calculating the probability of a sequence of events. The alignment is then produced in an optimisation framework such as maximum likelihood or Markov chain Monte Carlo by finding the set of events explaining the evolution of the sequences with a high probability and the parameters of the evolutionary model are estimated from the data. This approach is taken by computationally expensive methods such as BAli-Phy [19] and StatAlign [20] that integrate over all possible tree topologies. To make this more practical, FSA uses only pairwise comparisons in a statistical alignment framework and so reduces the running time drastically while sacrificing some of the accuracy [21].

In this paper, we introduce a novel corner-cutting method combined with progressive sequence alignment. Unlike former corner-cutting methods, our method does not define a compact part of the dynamic programming table to be filled in. The rationale behind the idea is the following. It is easy to see that any high-scored alignment is surrounded by a large set of low-scored alignments, and the number of low-scored alignments increases exponentially with the number of sequences. Indeed, a high-scored alignment contains several alignment columns containing homologous amino-acids. There are 2*^k ^*-2 ways to split an alignment column containing *k *characters into two columns with gaps. Any alignment containing such pair of alignment columns will be a neighbour of the high-scored alignment in the dynamic programming table. Furthermore, the score of these alignments will be Significantly lower than the score of the high-scored alignment, since the scores differ in two gap scores and the scores missing due to not aligning homologous amino-acids.

**Figure 1:**
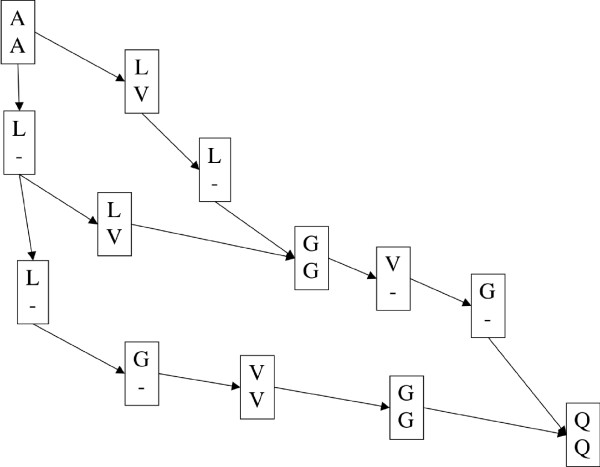
**Example alignment network**. This network shows three different alignments of the sequences ALLGVGQ and AVGQ.

**Figure 2:**
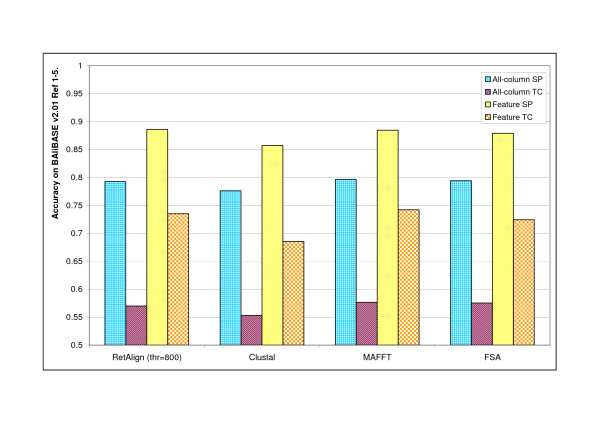
**Comparison of alignment software on BAliBASE v2.01 Refs **[1-5]. Alignment accuracy of multiple alignment programs compared to that of RetAlign as measured on BAliBASE v2.01 Reference sets [1-5] using the provided bali_score tool (SP and TC scores calculated on all of the columns versus on columns containing features are all shown). RetAlign was run with sequence weighting on, a single guide tree iteration and with a reticular threshold of 800. FSA was run in maximum sensitivity mode. MAFFT was run with the -auto switch and ClustalW with the default settings.

Instead of defining a compact part of the dynamic programming table, our approach stores a set of optimal and suboptimal alignments at each step of the progressive alignment procedure. At an internal node of the guide tree, the two sets of alignments of the two children nodes are aligned against each other. We use a special data structure for both representing the alignments and aligning the set of alignments against another set of alignments. The common parts of the alignments are represented only once, and aligned only once, thus saving a large amount of memory and running time. The alignments in our representation form a reticulated network (see for example Figure 1.), hence the name of the method. Previous works showed that the convex hull of the optimal and suboptimal alignments might be relatively large (see, for example, Figure 2. in [22]). The volume of this convex hull grows exponentially with the number of alignments. On the other hand, our method maintains only the reticulated network instead of the entire convex hull thus saving a large amount of memory.

The method has been implemented in the Java programming language, tested on the BAliBASE database [23], and compared with ClustalW [16], MAFFT [18] and Fast Statistical Alignment (FSA) [21]. Several scoring schemes have been implemented and assessed in the Reticular Alignment algorithm. We show that Reticular Alignment outperforms ClustalW even if a simple scoring scheme is applied. When sophisticated scoring models are applied (like sequence weighting in sum-of-pairs scoring, decreasing gap penalties for runs of hydrophilic amino-acids, etc.) Reticular Alignment outperformes FSA and even MAFFT in some accuracy measurement.

## Methods

In this section, we describe the algorithms and theorems which are the theoretical background of the Reticular Alignment algorithm.

### The Waterman-Byers algorithm and ***x***-networks

Let *A *and *B *be two sequences over an alphabet Σ, of lengths *n *and *m*, respectively. Let *A_i _*denote the *i *long prefix of sequence *A*, and let *A_i _*denote the suffix of *A *starting in the *i *+ 1*^st ^*position. In this way, *A_i _*◦ *A^i ^*= *A*, where ◦ denotes concatenation. Let *a_i _*denote the character of *A *in position *i*.

Let *s *: Σ × Σ → *R *be a similarity function. *g_o _*will denote the gap opening and *g_e _*will denote the gap extension penalty. The score of any alignment, and thus all introduced concepts based on the alignment scores depend on the choices on similarity function, gap opening and gap extension penalty. However, for sake of simplicity, we omit to denote this dependence.

The Waterman-Byers algorithm [24] produces all alignments that have a score no less than the score of the optimal alignment minus some constant value. Here we show a variant of the algorithm that our method is based on. The algorithm is built up of 3 parts: a forward-align algorithm, a backward-align algorithm, and the alignment search algorithm that finds all alignments above a given score using the scores calculated by the forward and backward algorithms.

The forward-align algorithm calculates the score of the best alignment of prefixes *A_i _*and *B_j _*within the following 3 subsets of alignments:

• alignments ending in two aligned (matched) characters. The score of the best alignment of prefixes *A_i _*and *B_j _*in this set is denoted by *M_f _*(*i*, *j*).

• alignments ending in an insertion of character *b_j_*. The score of the best alignment of prefixes *A_i _*and *B_j _*in this set is denoted by *I_f _*(*i*, *j*).

• alignments ending in a deletion of character *a_i_*. The score of the best alignment of prefixes *A_i _*and *B_j _*in this set is denoted by *D_f _*(*i*, *j*).

The score of the optimal alignment of prefixes *A_i _*and *B_j _*is then max{*M_f _*(*i*, *j*), *I_f _*(*i*, *j*), *D_f _*(*i*, *j*)}. *M_f _*, *I_f _*and *D_f _*can be efficiently calculated using dynamic programming. The DP tables are initialised as:

(1) $$M_{f}(0,0)=0$$

(2) $$I_{f}(0,0)=D_{f}(0,0)=-\infty$$

(3) $$M_{f}(i,0)=I_{f}(i,0)=-\infty \;i>0$$

(4) $$D_{f}(i,0)=g_{0}+(i-1)*g_{e}\;i>0$$

(5) $$M_{f}(0,j)=D_{f}(0,j)=-\infty \;j>0$$

(6) $$I_{f}(0,j)=g_{o}+(j-1)*g_{e}\;j>0$$

The dynamic programming recursion then goes from shorter prefixes towards larger prefixes in the following way:

(7) $$\begin{array}{l} M_{f}(i,j)=\max\{M_{f}(i-1,j-1),\\ I_{f}(i-1,j-1),D_{f}(i-1,j-1)\}+s(a_{i},b_{j}) \end{array}$$

(8) $$\begin{array}{l} I_{f}(i,j)=\max\{M_{f}(i,j-1)+g_{o},\\ I_{f}(i,j-1)+g_{e},D_{f}(i,j-1)+g_{o}\} \end{array}$$

(9) $$\begin{array}{l} D_{f}(i,j)=\max\{M_{f}(i-1,j)+g_{o},\\ I_{f}(i-1,j)+g_{o},\;D_{f}(i-1,j)+g_{e}\} \end{array}$$

The backward-align algorithm is more sophisticated. Let *a_i_/-_j _*denote the alignment column showing deletion of *a_i _*in an alignment in which the first character in sequence *B *to the left of this alignment column is *b_j_*. Similarly, let *-_i_/b_j _*denote the alignment column showing the insertion of character *b_j _*in an alignment in which the first character in sequence *A *to the left of this alignment column is *a_i_*. The backward algorithm calculates the following three types of entries:

1. *M_b_*(*i*, *j*) denotes the score of the best alignment of suffixes *A^i ^*and *B^j ^*whose score is calculated as if the *a_i_/b_j _*alignment column was before it. Namely, if the alignment starts with a gap, it will be scored with the gap opening penalty.

2. *I_b_*(*i*, *j*) denotes the score of the best alignment of suffixes *A^i ^*and *B^j ^*whose score is calculated as if the -*_i_/b_j _*alignment column was before it. Namely, if the alignment starts with an insertion, it will be scored with the gap extension penalty.

3. *D_b_*(*i*, *j*) denotes the score of the best alignment of suffixes *A^i ^*and *B^j ^*whose score is calculated as if the *a_i_/-j *alignment column was before it. Namely, if the alignment starts with a deletion, it will be scored with the gap extension penalty.

These backward alignment scores can also be computed using a dynamic programming approach similar to the forward case. The initialisation of the backward DP tables is:

(10) $$M_{b}(n,m)=I_{b}(n,m)=D_{b}(n,m)=0$$

(11) $$M_{b}(n-1,m)=I_{b}(n-1,m)=g_{o}$$

(12) $$\begin{array}{l} M_{b}(i,m)=I_{b}\left(i,m\right)=\\ g_{o}+(n-i-1)*g_{e}\;i<n-1 \end{array}$$

(13) $$D_{b}(i,m)=(n-i)*g_{e}\;i>0$$

(14) $$\begin{array}{l} M_{b}\left(n,j\right)=D_{b}\left(n,j\right)=\\ g_{0}+(m-j-1)*g_{e}\;j<m-1 \end{array}$$

(15) $$M_{b}(n,m-1)=D_{b}(n,m-1)=g_{o}$$

(16) $$I_{b}(n,j)=(m-j)*g_{e}\;j>0$$

The DP tables are then filled from the shorter suffixes towards the longer suffixes, that is, backward on the indices. The recursions:

(17) $$\begin{array}{l} M_{b}(i,j)=\max\{M_{b}(i+1,j+1)+s(a_{i+1},b_{j+1}),\\ I_{b}(i,j+1)+g_{o},\;D_{b}(i+1,j)+g_{o}\} \end{array}$$

(18) $$\begin{array}{l} I_{b}(i,j)=\max\{M_{b}(i+1,j+1)+s(a_{i+1},b_{j+1}),\\ I_{b}(i,j+1)+g_{e},\;D_{b}(i+1,j)+g_{o}\} \end{array}$$

(19) $$\begin{array}{l} D_{b}(i,j)=\max\{M_{b}(i+1,j+1)+s(a_{i+1},b_{j+1}),\\ I_{b}(i,j+1)+g_{o},D_{b}(i+1,j)+g_{e}\} \end{array}$$

Using the forward and the backward scores it is possible to find all alignment columns that appear in an alignment with a score above a given threshold. This is based on the following theorem:

#### Theorem 1

*The score of the best alignment containing alignment column a_i_/b_j _(or -_i_*,/*b_j_, a_i_*/-*_j_,respectively) is M_f _*(*i*, *j*) + *M_b_*(*i*, *j*) *(I_f _*(*i*, *j*) + *I_b_*(*i*, *j*), *D_f _*(*i*, *j*) + *D_b_*(*i*, *j*)*) ^.^*

#### Proof

We give a proof for the first case, the proof for the other two cases goes in the same way. If an alignment contains *a_i_*/*b_j_*, then cutting the alignment after this alignment column will create two alignments. The left one is an alignment of prefixes *A_i _*and *B_j _*in which the last alignment column is *a_i_*/*b_j_*. The right one is an alignment of suffixes *Ai *and *Bj *whose score is calculated by adding the alignment column *a_i_*/*b_j _*before it. The best scored alignment containing *a_i_*/*b_j _*are cut into the best scored left and right alignment, by definition, with scores *M_f _*(*i*, *j*) and *M_b_*(*i*, *j*). The score of the alignment is the sum of these two values.

Theorem 1 provides the means to collect the alignment columns that participate in an alignment having score above a given threshold. The best score of the alignment column will be denoted by *b*(*α*). We define the *x*-network of the alignments in the following way.

#### Definition

For any sequences *A *and *B*, *x *≥ 0, the ***x*-network **of the alignments of *A *and *B *is a directed graph *G*(*V*, *E*). The vertex set consists of alignment columns *α *for which *b*(*α*) ≥ *opt - x*, where *opt *is the score of the optimal alignment of *A *and *B*; plus two auxiliary vertices, representing the beginning and the end of the alignment. These two auxiliary vertices are denoted by *Start *and *End*. An edge is going from vertex *α*_1 _to vertex *α*_2 _if there is an alignment in which *α*_1 _is followed by *α*_2_. The outgoing edges from the *Start *vertex go to the alignment columns with which the alignment might start, and the incoming edges of the *End *vertex come from the alignment columns that might be at the end of an alignment.

The following theorem states that an *x*-network never contains dead ends.

#### Theorem 2

*For any sequences A, B, x *≥ 0, *and α vertex of the x-network, there is a directed path from Start to α and also from α to End*.

#### Proof

Since *α *is in the *x*-network, *b*(*α*) ≥ *opt - x*. Consider an alignment containing *α *with score *b*(*α*). Any *α' *of this alignment has a best score greater or equal than *b*(*α*), hence they are all in the vertex set of the *x*-network. This alignment defines one possible directed path from *Start *to *α *and also from *α *to *End*.

An *x*-network can be constructed using an algorithm that first runs the forward and backward algorithm to calculate *b*(*α*) for each possible alignment column *α*, selects those columns for which *b*(*α*) ≥ *opt - x*, and builds the network from them.

### Aligning a network of alignments to a network of alignments

We are going to extend the Waterman-Byers algorithm to align a network of alignments to another network of alignments. First we define the network of alignments.

#### Definition

A network of alignments of sequences *A*_1_, *A*_2_, . . . *A_k_*, *k *≥ 1 is a directed acyclic graph whose vertices are alignment columns of the set of sequences together with a unique source (denoted by *Start*) and a unique sink (denoted by *End*). The vertices along any path from the source to the sink form a multiple sequence alignment of the set of sequences.

Obviously, an *x*-network is a network of alignments. Moreover, any single sequence (meaning *k *= 1) can be considered a simple, formal network. In that case, the formal alignment columns contain only one character, and the network is a single line containing only one alignment. We can generalise the definition of the *x*-network of two sequences to the *x*-network of two networks of alignments. For this, we first have to define the alignment of alignments.

#### Definition

An alignment of two alignments A and ℬ of sequences *A*_1_, *A*_2_, . . . *A_k _*and *B*_1_, *B*_2_, . . . *B_l _*is a multiple sequence alignment of these *k *+ *l *sequences such that the non all-gap columns of the first *k *rows gives back A, and the non all-gap columns of the last *l *rows gives back ℬ.

When we take an alignment column containing all-gap characters in the first *k *rows or in the last *l *rows, we indicate what was the previous non all-gap alignment column from A or ℬ. For example, *^a^_i_/-_j _*indicates an alignment column in which the first *k *row is the *i^th ^*alignment column from A, the last *l *rows contain only gaps, and the first alignment column to the left of this alignment column in which the last *l *rows contain not only gaps is the *j^th ^*alignment column from ℬ. In the next definition when we talk about alignment columns from the multiple alignment of all *A*s and *B*s sequences, we always mean alignment columns containing this additional information. Similarly, from now on, we always assume that the alignment is an alignment of two alignments, the first containing *k *lines, the second containing *l *lines.

#### Definition

For any two networks of alignments **A **and **B **and *x *≥ 0, the *x*-network of **A **and **B **is a directed graph *G*(*V*, *E*). The vertex set consists of two auxiliary vertices, representing the beginning and the end of the alignment and all alignment columns *α *for which *b*(*α*) ≥ *opt - x*, where *b*(*α*) is the maximal score of the alignment that can be achieved by aligning an alignment A ∈ **A **to an alignment ℬ ∈ **B **so that it contains the column *α*. *opt *is the maximal score that can be achieved by aligning any alignment A ∈ **A **to any alignment ℬ ∈ **B**. An edge is going from *α*_1 _to *α*_2 _if there is an alignment in which *α*_1 _and *α*_2 _are neighbour columns. The outgoing edges from *Start *go to the vertices that might be the first alignment column in an alignment, and the incoming edges of the *End *vertex come from the vertices that might be the last alignment column in an alignment.

When we align a network to a network using a dynamic programming algorithm, it is important to visit the alignment columns of the network in an order such that the entries are already calculated by the time we want to use them in the dynamic programming recursion. Therefore we introduce the linear extension of networks that can be used for traversing the network.

#### Definition

A linear extension of a directed acyclic graph is a total ordering, <, on the vertices such that for any two vertices *v *and *u*, if there is a directed path from *v *to *u *then *v < u*.

Furthermore, the forward-align and the backward-align algorithms work with prefix-alignments and suffix-alignments defined in the following way.

#### Definition

A prefix-alignment is a prefix of an alignment achievable by aligning an alignment A ∈ **A **to an alignment ℬ ∈ **B**. Similarly, a suffix-alignment is a suffix of an alignment achievable by aligning an alignment A ∈ **A **to an alignment ℬ ∈ **B**.

The generalisation of the Waterman-Byers algorithm is the following. The input consists of a threshold value *x *≥ 0 and a couple of networks of alignments, **A **and **B**, together with a linear extension for each network. The output is the *x*-network of **A **and **B **together with a linear extension of it.

The algorithm uses a forward and a backward dynamic programming algorithm. The forward align algorithm calculates the score of the best prefix-alignment in which the last non all-gap columns in the first *k *lines is *a_i _*and in the last *l *lines is *b_j _*for each subset of alignments:

• alignments ending with *a_i_*/*b_j_*. The score will be denoted by *M_f _*(*i*, *j*).

• alignments ending with *-_i_*/*b_j_*. The score will be denoted by *I_f _*(*i*, *j*).

• alignments ending with *a_i_*/*-_j_*. The score will be denoted by *D_f _*(*i*, *j*).

The initialisation is:

(20) $$M_{f}(0,0)=0$$

(21) $$I_{f}(0,0)=D_{f}(0,0)=-\infty$$

The dynamic programming algorithm visits the vertices of the two networks in their linear order. The recursions are:

(22) $$\begin{array}{l} M_{f}(i,j)=\underset{i^{'}\in N^{+}(i)}{\max}\underset{j^{'}\in N^{+}(j)}{\max}\{M_{f}(i^{'},j^{'}),\\ I_{f}(i^{'},j^{'}),\;D_{f}(i^{'},j^{'})\}+s(a_{i},b_{j}) \end{array}$$

(23) $$\begin{array}{l} I_{f}(i,j)=\;\underset{j^{'}\in N^{+}(j)}{\max}\;\{M_{f}(i,j^{'})+g(a_{i},{-}_{i},b_{j^{'}},b_{j}),\\ I_{f}(i,j^{'})+g({-}_{i},{-}_{i},b_{j^{'}},b_{j}),\\ D_{f}(i,j^{'})+g(a_{i},\;{-}_{i},\;{-}_{j'},b_{j})\} \end{array}$$

(24) $$\begin{array}{l} D_{f}(i,j)=\;\underset{i^{'}\in N^{+}(i)}{\max}\;\{M_{f}(i^{'},j)+g(a_{i^{'}},a_{i},b_{j},{-}_{j}),\\ I_{f}(i^{'},j)+g({-}_{i^{'}},a_{i},b_{j},{-}_{j}),\\ D_{f}(i^{'},j)+g(a_{i^{'}},a_{i},{-}_{j},{-}_{j})\} \end{array}$$

where $N^{+}$(*i*) is the set of indices of vertices sending an edge to the vertex indexed by *i*, and *g*(*a*, *b*, *c*, *d*) is the gap penalty function for alignment column *b/d *preceded by alignment column *a*/*c*. We assume that the gap penalty for a given alignment column can be calculated from the alignment column in question and its preceding alignment column. See details in the subsection *Gap penalties *below. The maximum of an empty set is defined to be - ∞.

The backward algorithm calculates the following scores:

• *M_b_*(*i*, *j*) denotes the score of the best suffix-alignment that can follow the alignment column *a_i_*/*b_j_*. Furthermore, the gap score of the first alignment column is calculated as if *a_i_/b_j _*was inserted before the first alignment column.

• *I_b_*(*i*, *j*) denotes the score of the best suffix-alignment that can follow the alignment column *-_i_*/*b_j_*. Furthermore, the gap score of the first alignment column is calculated as if *-_i_*/*b_j _*was inserted before the first alignment column.

• *D_b_*(*i*, *j*) denotes the score of the best suffix-alignment that can follow the alignment column *a_i_*/*-_j_*. Furthermore, the gap score of the first alignment column is calculated as if *a_i_*/*-_j _*was inserted before the first alignment column.

The initialisation of the dynamic programming algorithm is

(25) $$\begin{array}{l} M_{b}(n,m)=I_{b}(n,m)=D_{b}(n,m)=0\\ \forall n\in N^{+}(End_{\text{A}}),\;m\in N^{+}(End_{\text{B}}) \end{array}$$

where $N^{+}$(*End***_A_**) and $N^{+}$(*End***_B_**) are the sink vertex of networks **A **and **B**, respectively.

The dynamic programming algorithm visits the vertices of the two networks backward in their linear extension. The recursions are

(26) $$\begin{array}{l} M_{b}(i,j)=\underset{i^{'}\in {\text{N}}^{-}(i)}{\max}\underset{j^{'}\in {\text{N}}^{-}(j)}{\max}\{M_{b}(i^{'},j^{'})\\ +s(a_{i^{'}},b_{j^{'}}),I_{b}(i,j^{'})+g(a_{i},{-}_{i},b_{j},b_{j^{'}}),\\ D_{b}(i^{'},j)+g(a_{i},a_{i^{'}},b_{j},{-}_{j})\} \end{array}$$

(27) $$\begin{array}{l} I_{b}(i,j)=\underset{i^{'}\in {\text{N}}^{-}(i)}{\max}\underset{j^{'}\in {\text{N}}^{-}(j)}{\max}\{M_{b}(i^{'},j^{'})\\ +s(a_{i^{'}},b_{j^{'}}),I_{b}(i,j^{'})+g({-}_{i},{-}_{i},b_{j},b_{j^{'}}),\\ D_{b}(i^{'},j)+g({-}_{i},a_{i^{'}},b_{j},{-}_{j})\} \end{array}$$

(28) $$\begin{array}{l} D_{b}(i,j)=\underset{i^{'}\in {\text{N}}^{-}(i)}{\max}\underset{j^{'}\in {\text{N}}^{-}(j)}{\max}\{M_{b}(i^{'},j^{'})\\ +s(a_{i^{'}},b_{j^{'}}),I_{b}(i,j^{'})+g(a_{i},{-}_{i},{-}_{j},b_{j^{'}}),\\ D_{b}(i^{'},j)+g(a_{i},a_{i^{'}},{-}_{j},{-}_{j})\} \end{array}$$

where $N^{-}$(*i*) is the set of indices of vertices to which an edge is going from the vertex with index *i*. Similarly to Theorem 1., it is true that the best score of alignments containing *a_i_*/*b_j_*, *-_i_*/*b_j _*and *ai*/*-_j _*is *M_f _*(*i*, *j*) + *M_b_*(*i*, *j*), *I_f _*(*i*, *j*) + *I_b_*(*i*, *j*) and *D_f _*(*i*, *j*) + *D_b_*(*i*, *j*), respectively. Therefore, the pair of indices (*i*, *j*) is visited in lexicographical order, and those alignment columns *α *= *a_i_*/*b_j _*or *-_i_*/*b_j _*or *ai*/*-_j _*are selected for which *b*(*α*) ≥ *opt -x*. The maximal score, *opt*, can be calculated from the following equation

(29) $$\begin{array}{l} opt=\underset{n\in N^{+}(End_{\text{A}})}{\max}\underset{m\in N^{+}(End_{\text{B}})}{\max}\{M_{f}(n,m),\\ I_{f}(n,m),D_{f}(n,m)\} \end{array}$$

Similarly to Theorem 2., it is easy to show that there are no dead ends in the so constrained network. The following theorem states that visiting the alignment columns in lexicographical order will provide a linear extension for the constructed *x*-network.

#### Theorem 3

*The lexicographical ordering of alignment columns together with arbitrary ordering of a_i_*/*b_j_, -_i_*/*b_j _and a_i_*/*_-j _is a linear extension for the x-network of networks ***A ***and ***B ***if the indices i's and j's are linear extensions for the networks ***A ***and ***B**, *respectively*.

#### Proof

The preceding alignment columns for *a_i_*/*b_j _*might be *a_i'_/b_j'_*, *-_i'_/b_j' _*or *a_i'_/-_j' _*for some *i' *∈ $N^{+}$(*i*) and *j' *∈ $N^{+}$(*i*). Since indices in **A **are linear extensions, *i' < i *for any *i' *∈ $N^{+}$(*i*), and thus, in the lexicographical order, all possible preceding alignment columns are smaller than *a_i_*/*b_j_*^.^

The preceding alignment columns for *-_i_*/*b_j _*might be *a_i_/b_j'_*, *-_i_/b_j' _*or *a_i_/_j' _*for some *j' *∈ $N^{+}$(*j*). Since indices in **B **are linear extensions, *j' < j *for any *j' *∈ $N^{+}$(*j*), and thus, in the lexicographical order, all possible preceding alignment columns are smaller than *-_i_*/*b_j_*

The preceding alignment columns for *a_i_/-_j _*might be *a_i'_/b_j_*, *-_i'_/b_j _*or *a_i'_/-_j _*for some *i' *∈ $N^{+}$(*i*). Since indices in **A **are linear extensions, *i' < i *for any *i' *∈ $N^{+}$(*i*), and thus, in the lexicographical order, all possible preceding alignment columns are smaller than *a_i_/-_j_*.

### The Reticular Alignment algorithm

The Reticular Alignment algorithm is the following:

1. Build or load a guide tree for the sequences

2. Transform the sequences at the leaves of the guide tree into simple 'linear' networks

3. Visit the internal nodes of the guide tree in reverse traversal order. For each internal node *v *with children *u*_1 _and *u*_2_, labelled with the networks of alignments **A_1 _**and **A_2_**, respectively, calculate the *x_v_*-network of **A_1 _**and **A_2 _**using the generalised Waterman-Byers algorithm

4. Return the best scored alignment from the *x*-network calculated at the root of the guide tree.

When *x *is set to 0, only the (locally) optimal multiple alignments are stored in the *x*-network. In this case, the Reticular Alignment algorithm mimics a standard progressive alignment method. When *x *is set to ∞, the Reticular Alignment method performs an exhaustive search in the space of multiple alignments, namely, it finds the best scored alignment. As *x *increases, the size of the network also increases, having a similar effect on the running time and memory usage. Along with the *x *value the Reticular Alignment algorithm can be parameterised in a list of ways:

• guide tree construction method

• similarity scoring of alignment columns

• gap scoring model and gap penalties

• strategy to select threshold values at the internal nodes

Here we briefly describe the choices we had and the decisions we made considering these aspects of the algorithm.

#### Building the guide tree

Standard methods for constructing a guide tree using pairwise comparisons of the input sequences include UPGMA and Neighbour Joining (NJ) [25,26]. We implemented both and allow the user to choose between the two algorithms or to provide their own guide tree.

The NJ algorithm generates an unrooted tree. Because RetAlign requires a rooted tree that can be traversed from the leaves upwards, we root the tree using the 'mid-point' method as described in [16]. The computationally most expensive step of the guide tree construction process is the calculation of the pairwise distances between the sequences. For increased accuracy, we opted to perform a full dynamic programming alignment between each pair of sequences and transform the similarity scores into distances using the formula:

(30) $$D_{ij}=S_{ii}+S_{jj}-2S_{ij}$$

In a future version of RetAlign we plan to implement a basic optimisation such as the Hirschberg algorithm [27] to reduce the memory usage of this initial alignment phase from Θ(*L*^2^) to Θ(*L*) where L is length of the longest sequence.

#### Gap penalties

It is well known that affine gap penalties (having gap opening and gap extension penalties) generate Significantly more accurate pairwise alignments than linear gap penalties. Therefore it seems reasonable to define similar gap penalties for multiple sequence alignments. One natural way is the sum-of-pairs scoring with affine gap penalty, when one generates all pairwise alignments from the multiple alignment, removes all-gap columns, scores the so-obtained alignment using affine gap penalty, and sums these scores over all pairwise alignments. Surprisingly, finding the best sum-of-pairs scored multiple alignment between two multiple alignments is NP-complete [28]. The heuristic explanation is that the question whether a gap-opening or a gap-extension penalty should be calculated for an alignment column can be answered only after removing the all-gap alignment columns from the pairwise alignment taken from the multiple alignment. Rarely can the question be answered by looking back at the previous alignment column only. The exact sum-of-pairs scoring problem is generally hard to solve, but in some cases it can be solved unambiguously by looking back at the previous alignment column. Furthermore, it can always be solved this way when aligning only two sequences. We developed a gap scoring scheme that approximates the sum-of-pairs gap score and can be calculated efficiently by looking at adjoining alignment columns only. We assign a score to each combination of patterns that any two rows from two adjoining columns can form. These scores then need to be summed for all sequence (row) pairs to obtain the indel score for the two columns. The full indel score for a multiple alignment is then the sum of the indel scores of the consecutive alignment columns. The indel matrix on Table 1. shows the score value used for each pattern combination. The goals in mind when filling up this matrix were to make the resulting scoring

**Table 1:** Insertion-deletion score matrix used by RetAlign.

	- -	- *	* -	* *
- -	-	*OP/*2	*OP/*2	*EX*
- *	*OP/*2	*-*	*OP*	*OP/*2
* -	*OP/*2	*OP*	-	*OP/*2
* *	*EX*	*OP/*2	*OP/*2	-

Row: indel pattern formed by two consecutive columns of the 1*^st ^*sequence, column: that of the 2^*nd *^sequence. *OP *and *EX *are the gap opening and extension penalty.

• *consistent *in that it does not depend on the order of the sequences within the selected pairs (the indel matrix is symmetric)

• *symmetric *- the reverse of a multiple alignment has the same score (the score of a pattern and its horizontally flipped variant is the same)

• best approximate the sum-of-pairs scores

The simplest case to consider is when there is an insertion in one of the sequences: $\begin{array}{ccccc} {}* & - & - & - & *\\ {}* & * & * & * & * \end{array}$

The sum-of-pairs indel score of this alignment is *OP *+ 2*EX *where *OP *and *EX *are the gap opening and extension penalty. This - and any similar cases where the length of the insertion is different - can be mimicked precisely if (and only if) the score of the pattern $\begin{array}{cc} - & -\\ {}* & * \end{array}$ is *EX *while the score of $\begin{array}{cc} {}* & -\\ {}* & * \end{array}$ and $\begin{array}{cc} - & *\\ {}* & * \end{array}$ is both *OP/*2 due to the symmetric property. The score of $\begin{array}{cc} {}* & -\\ - & * \end{array}$ is *OP *for similar reasons as it starts a new sequence of gaps in both directions. With this choice cases such as $\begin{array}{cccccc} {}* & - & - & * & * & *\\ {}* & * & * & - & - & * \end{array}$ are handled properly. To avoid Significant overestimation of the score of $\begin{array}{ccccc} {}* & - & - & - & *\\ {}* & - & - & - & * \end{array}$ which is 0 in the sum-of-pairs scheme, a score of 0 must be assigned to both $\begin{array}{cc} - & -\\ - & - \end{array}$ and $\begin{array}{cc} - & *\\ - & * \end{array}$ Then the only pattern left to assign a score to is $\begin{array}{cc} {}* & -\\ - & - \end{array}$ (and its 3 mirror images). The problem with this one is that the score should depend on whether the gap in $\begin{array}{c} {}*\\ - \end{array}$ is extended in the next non-gap-only column to the right. The three possibilites are (1) $\begin{array}{cccc} {}* & - & - & *\\ - & - & - & - \end{array}$, (2) $\begin{array}{cccc} {}* & - & - & -\\ - & - & - & * \end{array}$ and (3) $\begin{array}{cccc} {}* & - & - & *\\ - & - & - & * \end{array}$ so it is easy to see that a score of *EX/*2 suffices for (1) and *OP/*2 does for (2,3) (note that the pattern in question repeats twice in (1,2) so the total scores of *EX*, *OP *and *OP/*2 are obtained that match the scores of the corresponding patterns formed by removing the gap-only columns). We resolved the ambiguity by choosing *OP/*2 as the score because we expected this to provide the best approximation of the sum-of-pairs scores with a systematic overscoring in cases such as (1). Other alternatives include *EX *or *EX/*2, both of which have been later shown to yield slightly lower overall accuracy as measured on the BAliBASE reference database.

In addition to RetAlign's default pairwise indel score model presented above we also implemented the simplified, non-pairwise indel scoring method used in ClustalW. In this scheme, when two sets of sequences are aligned, each insertion or deletion of a full alignment column receives a single gap penalty - even if the alignment column contains several gaps. This score can be computed considerably faster (although we implemented tricks allowing the calculation of the pairwise indel scores in linear time in the number of sequences) but creates anomalies when suboptimal alignments are inserted or deleted: the gaps 'hidden' in the columns of the suboptimal alignments are not penalised and these columns become overly represented in the final alignment (see results). Unlike the pairwise indel scoring this score cannot be used as an accuracy measure of multiple alignments because it depends on which sets of sequences are being aligned in the last step.

#### Scoring similarities

We score substitutions in accordance with the sum-of-pairs scoring scheme. A similarity score is computed for each alignment column as the sum of similarity values for each pair of non-gap characters in the column (in all experiments, the BLOSUM62 matrix was used for scoring pairwise character similarities [29]). The similarity score is also computed for columns where an insertion or deletion occurs and creates a stack of gaps in the ongoing alignment step. The total similarity score of an alignment is simply the sum of similarity scores for all columns.

This pairwise scoring method is slightly different from ClustalW's approach where the substitution score is dependent on what the two sets of sequences are that are being aligned: only 'cross-scores' are taken into account (scores for pairs of non-gap characters where the first element of the pair is from a sequence in the first set and the second from the second set). The similarity score of columns with insertion or deletion in the ongoing alignment is thus zero. We also implemented this modified similarity scoring method and combined it with the non-pairwise indel scoring shown in the previous section to imitate ClustalW's scoring model.

#### Internal score and sequence weighting

We introduced the indel and similarity scoring models of RetAlign in the last two sections. The total (internal) score of a multiple alignment as produced by RetAlign is the sum of the indel and similarity scores, both of which are calculated pairwise. Though very similar, this score slightly deviates from the sum-of-pairs score as a result of the approximation of the indel score using adjoining alignment columns only - this is explained in detail above. In practice we did not encounter any situation where the difference was Significant and the optimisation targeted to maximise the internal score efficiently boosted the sum-of-pairs score, too. Both scores are completely independent from the phylogenetic tree connecting the sequences and can be used as an accuracy measure.

One inherent weakness of the sum-of-pairs scoring, though, is that evolutionary events separating a distant sequence from a number of closely related (overrepresented) sequences are overscored - one evolutionary event in time might be penalised several times in the alignment score. To overcome this, we introduced sequence weighting, based on principles set out in ClustalW. First, a list of weights is calculated and assigned to sequences using the topology and edge lengths of the guide tree, precisely as described in [16]. The weight of a sequence is calculated from the edge lengths of the branches leading to the sequence from the root node, and edge lengths of branches that are shared by two or more sequences are divided up equally between them. The weights are the sums of these partial lengths. Once the weights are available, the pairwise scoring method can be applied with the modification that whenever a score is calculated for a pair of sequences it is multiplied by both sequence weights. The sum of these weighted scores gives rise to an overall score that is much less biased by the overrepresented sequences.

#### Threshold values

The size of the alignment space that is being explored by the Reticular Alignment algorithm and consequently, the accuracy of the alignments created depends on the strategy for choosing an *x *value for each alignment step at the internal nodes. We chose to set the *x *value dynamically such that the final size of the alignment network is at most (*t *+ 100)% of the length (number of columns) of one of the best scored multiple alignments, where *t *is a threshold parameter set by the user. Note that this is the "Threshold to be passed for computation" value on the GUI of our application. The slider underneath is just for convenience, with a *log *transformation. For any such *t *value, the corresponding *x *value can be found by building the network gradually: at first, alignment columns are placed in a priority queue where the key is the score of the best alignment they appear in; then groups of columns having equal score are removed from the queue iteratively, starting with the ones having the highest score, and added to the growing network *en masse *while the size limit permits.

This approach is more advantageous than if the *x *was constant or set to a fixed proportion of the optimum score because the later would have an unpredictable effect on the running time and memory usage and could also cause the alignment networks to vary considerably in relative size at the internal nodes. In contrast, with our method, the memory requirement can be estimated from *t *and the proportion of the number of suboptimal alignment columns to optimal columns is uniform over the whole tree.

#### Efficient score calculation

The scoring scheme of RetAlign involves summing similarity values and gap penalties for all sequence pairs. These pairwise summations are carried out repeatedly on alignment columns (for similarity) and pairs of columns (for gap scores) to fill each element of the dynamic programming tables. The straightforward implementation can thus have a huge impact on the running time when many sequences are aligned. For this reason we developed techniques to speed up these calculations.

The two problems are essentially the same: given a list of pattern values *v*_1_, . . . *v_n_*, pairwise pattern scores must be summed for all pairs: $S=\sum_{i=1}^{n}\sum_{j=1}^{n}M(v_{i},v_{j}),\;v_{i}\in \;\{p_{1},\ldots p_{k}\}$. In the similarity score case, the patterns are the residues (of 20 different types when aligning proteins) and the matrix is the similarity matrix, while in the indel score case there are 4 different patterns formed by two successive characters, both either gap or non-gap. The trick is simple: first count how many of each pattern type is present in the column, then sum the score value for the pair of types multiplied by the counts:

$c_{j}=\sum_{i=1}^{n}\delta_{v_{i},pj}(j=1,\ldots k)$ so that now $S=\sum_{i=1}^{k}\sum_{j=1}^{k}c_{i}c_{j}M(p_{i},p_{j})$. One distinct bonus of the idea is that it can be naturally adapted to sequence weighting: if *w*_1_, . . . *w_n _*are given in addition to the patterns then $S^{w}=\sum_{i=1}^{n}\sum_{j=1}^{n}w_{i}w_{j}M(v_{i},v_{j})$ can be calculated as $S^{w}=\sum_{i=1}^{k}\sum_{j=1}^{k}s_{i}s_{j}M(p_{i},p_{j})$ where $s_{j}=\sum_{i=1}^{n}w_{i}\delta_{v_{i},pj}(j=1,\ldots k)$ I.e. the count for each pattern type must simply be substituted by the sum of sequence weights for patterns of each type to obtain the scoring scheme with sequence weighting. It is an important implementation consideration how the list of counts or weight sums is represented. For the indel case, we opted to use an array of fixed length of 4 that means the calculation of a column score requires *n *+ 16 steps. In the similarity score case, however, we chose to store a list of (character, count/weight sum) pairs so that the calculation takes only *n *+ *l*^2 ^steps where *l *is number of different character types present in the column. Though this requires one or two additional table lookups per iteration, the savings are huge when there are conserved columns made up of just a few different characters.

## Results and Discussion

We implemented the Reticular Alignment method in the Java programming language with all features for the choices of parameters as described in the previous section. The method was tested on the BAliBASE database [23], and compared with ClustalW [16], MAFFT [18] and Fast Statistical Alignment (FSA) [21]. BAliBASE is a database of manually-refined multiple sequence alignments specifically designed for the evaluation and comparison of multiple sequence alignment programs. The alignments are categorised by sequence length, similarity, and presence of insertions and N/C- terminal extensions. Core blocks are identified excluding non-superposable regions.

BAliBASE provides a scoring tool (bali_score.c) to measure the accuracy of sequence alignments based on the reference alignments in the database. This tool offers two accuracy measures (SP and TC) and allows assessment based on either all or a subset of alignment columns thus essentially giving four different accuracy scores. SP is the number of correctly aligned residue pairs divided by the number of aligned residue pairs in the reference alignment, TC is the number of correctly aligned columns divided by the number of columns in the reference alignment. SP and TC can also be calculated on columns of the core blocks only - these feature columns are described in the BAliBASE database by separate files. We denote the so-obtained scores 'Feature SP' and 'Feature TC'. All four of these scores can be regarded as a sensitivity measure (in classiffcation terminology) because characters/columns incorrectly shown homologous do not decrease the score.

**Figure 3:**
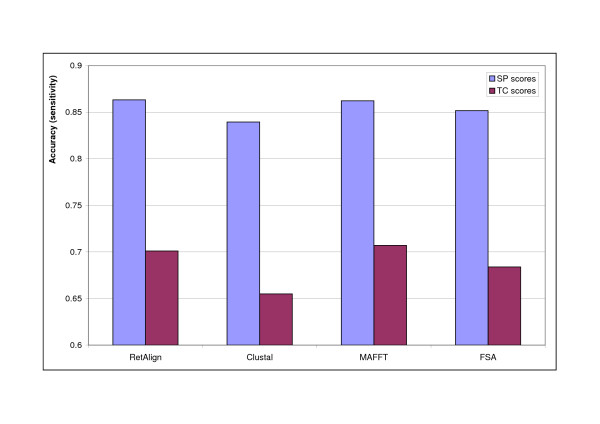
**Comparison of alignment software on BAliBASE v1.0**. Alignment accuracy of multiple alignment programs compared to that of RetAlign as measured on BAliBASE v1.0 using the provided bali_score tool (SP and TC scores are both shown). RetAlign was run with sequence weighting on, a single guide tree iteration and with a reticular threshold of 200. FSA was run in maximum sensitivity mode. MAFFT was run with the -auto switch and ClustalW with the default settings.

**Figure 4:**
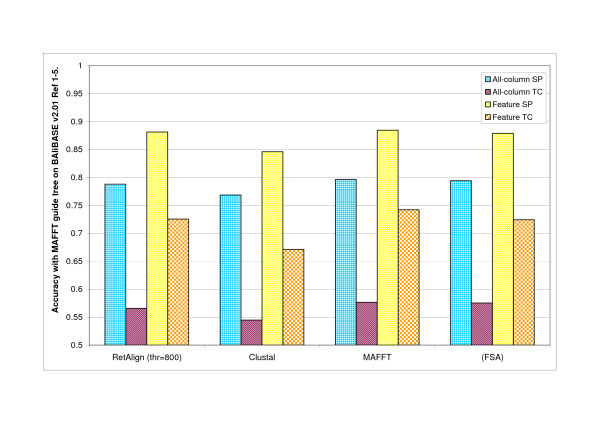
**Comparison of alignment software with fixed guide tree on BAliBASE v2.01 Refs **[1-5]. Alignment accuracy of multiple alignment programs compared to that of RetAlign as measured on BAliBASE v2.01 Reference sets [1-5] when guide tree is fixed to MAFFT's output. Note that FSA does not need a guide tree but accuracy figures are shown for convenience. RetAlign was run with sequence weighting on, a single guide tree iteration and with a reticular threshold of 800. FSA was run in maximum sensitivity mode. MAFFT was run with the -auto switch and ClustalW with the default settings.

We measured the accuracy of the Reticular Alignment method on BAliBASE v1.0 and v2.0 Ref1-5 datasets and compared it to the performance of the well-known alignment software ClustalW, MAFFT and FSA. See results in Figure 2 and 3. To separate the effect of the guide tree and allow a fair comparison of the alignment strategies we re-run ClustalW and RetAlign with the guide tree fixed to the one created by MAFFT. Results are shown in Figure 4.

We were interested in how the accuracy of our method depends on different parameters. Since the parameter space is four dimensional (guide tree building, similarity scoring, gap scoring, threshold value in the generalised Waterman-Byers algorithm) with several choices along each dimension, we do not show the results for each possible combinations of parameters. Two parameters (similarity scores, gap scores) influence only the score of the multiple alignments, one parameter (threshold value for the generalised Waterman-Byers algorithm) influences how to search in the search space, and one parameter (how to build the guide tree) influences the search strategy (when the tree-constructing strategy changes the topology of the tree, since our method is a progressive alignment method), and might also influence the way of scoring alignments (if the sequences are weighted by the guide tree). For each fixed score function, we tested how the alignment accuracy changes with the *t *parameter, namely, how much the accuracy can be improved by a deeper search in the alignment space. Some of our findings are quite surprising, discussed in the following subsections.

### Alignment accuracy generally increases with the deepened search in the alignment space

**Figure 5:**
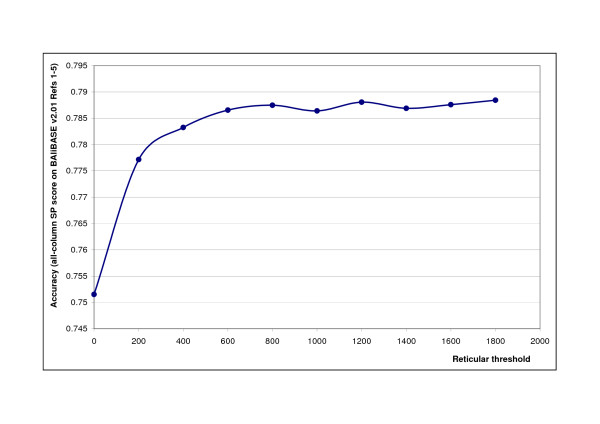
**Dependency of alignment accuracy (all-column SP) on the reticular threshold**. Alignment accuracy achieved by RetAlign for different reticular threshold values. Accuracy here is measured as the mean all-column SP score on BAliBASE v2.01 Reference sets [1-5]. RetAlign was run with sequence weighting on and pairwise indel scoring.

**Figure 6:**
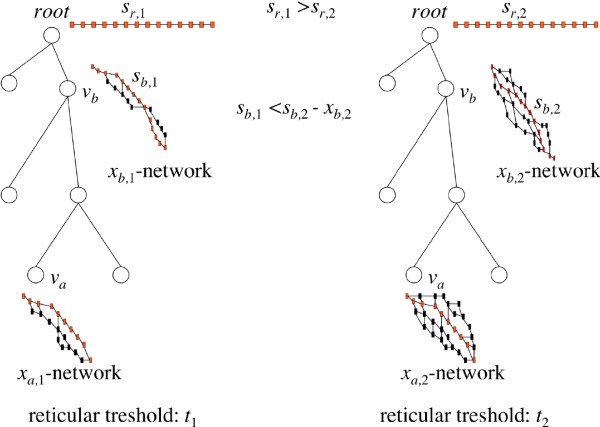
**Explaining how the internal score might decrease with the reticular threshold**. See text for details.

As the main novelty of our method is the sophisticated search for the best scored alignment, we first show the effect of the *t *parameter on the alignment accuracy. The average alignment accuracy improves as the *t *parameter increases, see Figure 5. However, this increase is not monotonous. There might be two reasons why a widened search may yield worse alignments. The first reason is simple: the better scored alignments are less accurate, hence, although the wider search found better scored alignments, these alignments agree less with the BAliBASE benchmark. The second reason is more sophisticated, and to understand this, the reader must have in mind that the globally optimal alignment might be achived via suboptimal solutions during the progressive alignment method. Having said this, imagine the following situation (see also Figure 6.): at a given reticular threshold value *t*_1_, the best alignment at internal node *v_a _*has a score *s_a_*, and an *x_a_*,1-network is generated at *v_a_*. From this network, the best alignment at some ancestral node of *v_a_*, denoted by *v_b _*has a score *s*_*b*,1_, and an *x_b_*,_1_-network is generated at *v_b_*. From this network, the best alignment at the root of the guide tree has score *s*_*r*,1_. Now we change the threshold to *t*_2_. The best alignment at node *v_a _*is still the same, but we create a larger, *x*_*a*,2_-network at this node, *x*_*a*,2 _>*x*_*a*,1_. From this network, we are able to find a better scored alignment at node *v_b_*, which has score *s*_*b*,2 _>*s*_*b*,1_. We build up an *xb*,2-network around this node, but it might happen that *s*_*b*,2 _- *x*_*b*,2 _>*s*_*b*,1_. This means that the new *x*_*b*,2_-network does not contain *any *alignment from the old, *x*_*b*,1_-network! Therefore, the progressive alignment method with reticular threshold *t*_2 _will operate on a set of alignments above the node *v_b _*which are completly different from the set of alignments appeared during the progessive alignment method with reticular threshold *t*_1_. The consequence is that the best alignment at the root obtained from the *x*_*b*,2_-network might have a score *s*_*r*,2 _<*s*_*r*,1_. Namely, the score of the final alignment might decrease with increasing the reticular threshold parameter.

**Figure 7:**
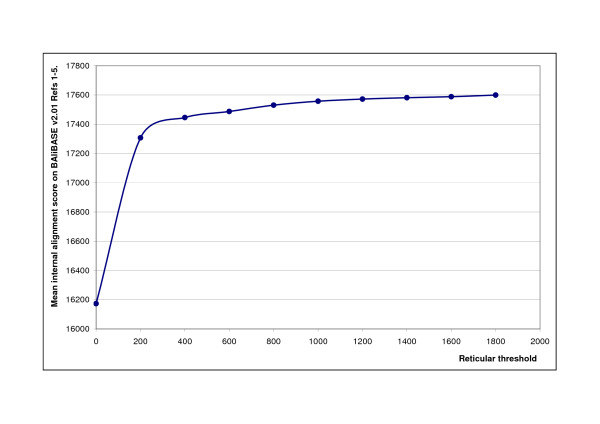
**Dependency of internal score on the reticular threshold**. Best internal alignment score achieved by RetAlign for different reticular threshold values. Score is the mean internal score on BAliBASE v2.01 Reference sets [1-5]. RetAlign was run with sequence weighting on and pairwise indel scoring.

To test the second hypothesis, the internal score of the alignments were measured, i.e. the score that the Reticular Alignment algorithm was to maximise. The dependency of this internal score on the *t *threshold value is shown on Figure 7. On average, this internal score is monotonously increasing, although we did find example sets of sequences for which the internal score decreased by increasing *t*. However, these examples were relatively rare. Hence, the slight occasional decrease in the accuracy caused by the increase of *t *is mainly due to the non-perfect correlation between the RetAlign's internal score of an alignment and the alignment accuracy measured on BAliBASE. The most interesting case is discussed in the next subsection.

### Comparing single and pairwise gap penalties

Clustal uses a simple non-pairwise gap-penalty for multiple alignments as described in the Methods section. This seems a rational choice for Clustal, as this gap scoring scheme indeed generates better alignments for Clustal than the pairwise scoring scheme.

**Figure 8:**
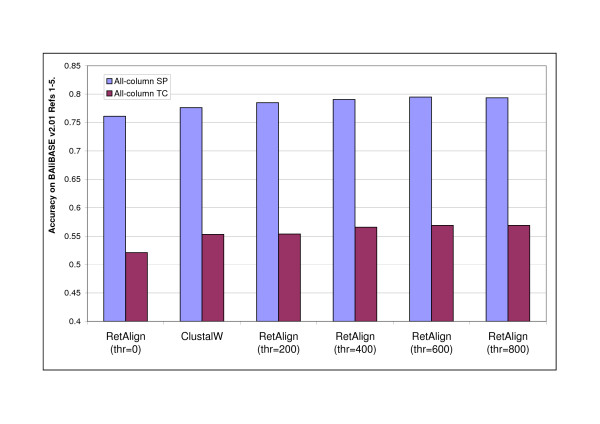
**Effect of pairwise indel scoring combined with reticular optimization**. Alignment accuracy of RetAlign for different reticular threshold values with only pairwise indel scoring enabled and comparison to that of ClustalW. With no other advanced scoring techniques RetAlign slightly outperformed ClustalW for thresholds over 200. Alignment accuracy was measured as the mean all-column SP and TC score on BAliBASE v2.01 Reference sets [1-5].

However, when we extend the scope of the search in the alignment space, and keep not only the locally optimal alignment during the progressive alignment procedure, we see a different picture. Increasing the *t *parameter when the alignments are scored using a pairwise gap penalty scheme yielded an increase in the accuracy of the generated alignments, and eventually the Reticular Alignment method with this gap-penalising scheme overtakes ClustalW, see Figure 8. We would like to highlight that in this experiment no further tricks were used by Reticular Alignment, like differentiating the gap penalties for hydrophobic and hydrophilic amino acids.

**Figure 9:**
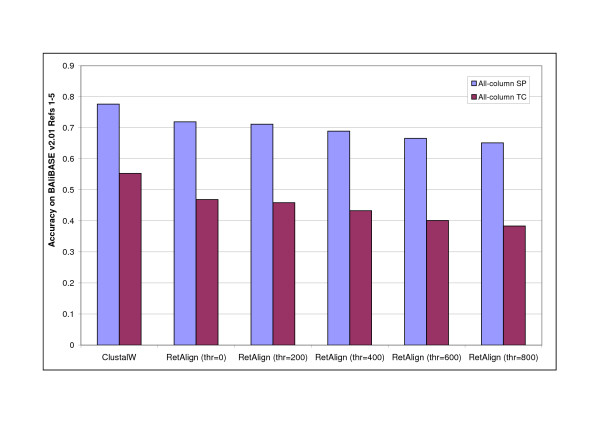
**Alignment accuracy with simple non-pairwise indel scoring**. Alignment accuracy of RetAlign for different reticular threshold values when indel scoring is non-pairwise. Accuracy monotonously decreases with the threshold and is always below that of ClustalW. Alignment accuracy was measured as the mean all-column SP and TC score on BAliBASE v2.01 Reference sets [1-5].

**Figure 10:**
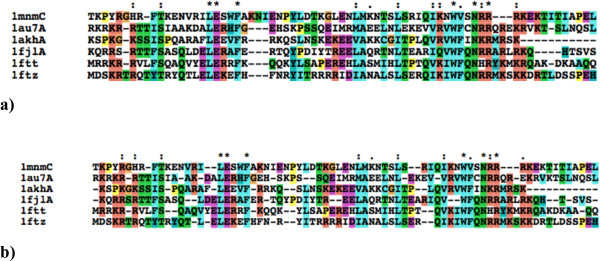
**Counterintuitive example for simple non-pairwise indel scoring**. An example showing why the accuracy of alignments might decrease with increasing the reticular threshold when the gaps are scored with simple non-pairwise indel scoring. **a) **The best alignment found with *t *= 0 threshold and simple non-pairwise indel scoring. Only the locally optimal alignments were kept in the progressive alignment, thus the final multiple alignment contains a few, aggregated gaps, for which the pairwise alignments are also optimal. **b) **The best alignment found with *t *= 800 threshold value and simple non-pairwise indel scoring. This alignment scored better than the previous alignment, because it contains more homologous pairs. The increased number of homologous amino acid pairs is achieved by inserting more gaps, however, the increased number of gaps do not reduce too much the score as simple non-pairwise indel scoring is applied. ClustalW never considers this alignment as it can be built only via sub-optimal alignments.

On the other hand, when simple non-pairwise gap penalties were applied, the accuracy decreased with increasing the *t *parameter, see Figure 9. A detailed analysis revealed that the internal score of the Reticular Alignment increased in this experiment. Namely, the method found better-scored alignments with increasing the explored search space, however, these better scored alignments are less accurate according to the BAliBASE database. However, these overall better scored solutions can only be constructed via locally suboptimal solutions, see Figure 10. Clustal does not consider suboptimal solutions, that is why it does not find these alignments, and thus generates worse-scored, on the other hand, more accurate alignments. This example clearly shows that the parameterisation problem is at least as important in the multiple sequence alignment than the optimisation problem.

### The importance of a good guide tree, sequence weighting and gap scoring

Although Reticular Alignment outperformed ClustalX with a simple sum-of-pairs scoring scheme, and without any sophisticated gap scoring scheme, its performance with the less sophisticated scoring schemes was worse than the performance of the cutting-edge multiple sequence alignment methods. Therefore, we improved the scoring scheme both for similarity scoring and for gap scoring.

It is well-known that the relative difference between the score of the fully conserved alignment column and the score of the alignment column with a single mismatch decreases with the number of sequences [30]. This artefact can be reduced by weighting the sequences according to the evolutionary tree showing their relationship. Such a weighting also improves alignment accuracy [30]. We implemented the same sequence weighting method that ClustalX uses.

Since our sequence weighting method uses the guide tree, it is also important to construct a good guide tree. We found that NJ outperforms UPGMA measured in alignment accuracy (data not shown). Since the NJ algorithm generates an unrooted tree, and the Reticular Alignment method needs a rooted tree, the NJ tree must be rooted. Changing the root of the guide tree also changes the progression of the multiple alignment. The more balanced the tree, the closer the numbers of sequences in the two alignment networks. We found that balanced trees generated by the 'mid-point' method as described in [16] generates more accurate alignments than unbalanced trees where one of the subtrees of the root contains only a single sequence.

Finally, it is also important to distinguish gap scores based on whether hydrophilic or hydrophobic amino-acids are inserted and/or deleted. Applying the same scoring scheme that ClustalX uses improved the alignment accuracy.

Fortifying the Reticular Alignment method with these sophisticated scoring schemes yielded a method that generated highly accurate alignments. Reticular Alignment outperformed all of ClustalX, MAFFT and FSA in SP values on the BAliBASE v1.0 database, and only MAFFT outperformed Reticular Alignment in the TC values, see Figure 3. On BAliBASE v2.0., Reticular Alignment outperformed ClustalX and FSA in all accuracy measurements, and it had a higher feature SP value than MAFFT, see Figure 2.

### Memory and Computational demand

**Figure 11:**
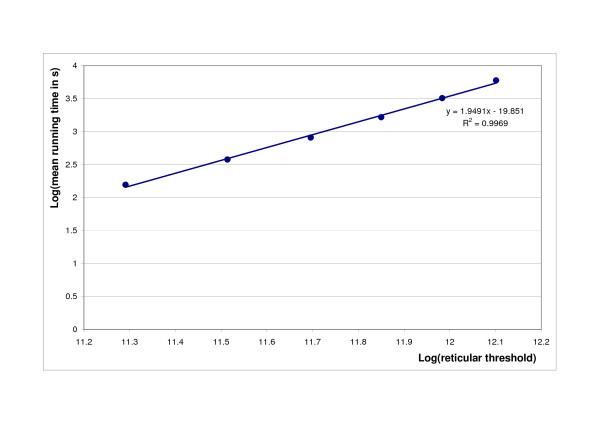
**Execution time growth rate with the reticular threshold**. Average running time of RetAlign on BAliBASE v2.01 Reference sets [1-5] for different reticular threshold values. Log-log scale is used to illustrate growth rate. RetAlign was run with sequence weighting on and pairwise indel scoring.

**Figure 12:**
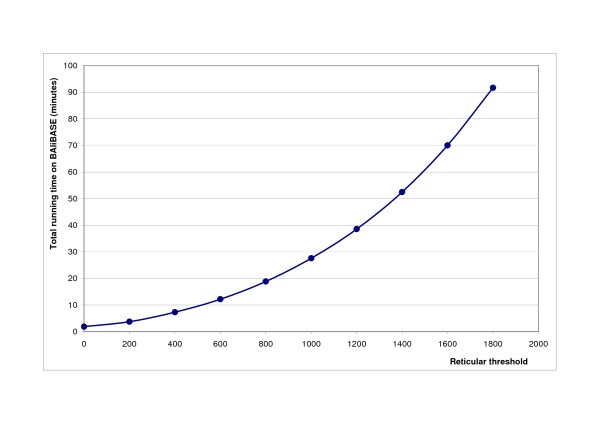
**Absolute execution time growth with the reticular threshold**. Total time required to run RetAlign on BAliBASE v2.01 Reference sets [1-5] for different reticular threshold values. RetAlign was run with sequence weighting on and pairwise indel scoring.

As the threshold value increases, the size of the alignment network will increase, too. Figure 11 and 12. show the dependence of running time on the reticular threshold value. The log-log scale plot in Figure 11. clearly indicates that the empirical running time grows quadratically with the reticular threshold. This agrees well with the theoretical considerations that the time required to align two alignment networks is proportional to the product of the two network sizes. The memory usage is also quadratic with the threshold value in the current implementation (data not shown), which restricts the applicability of the software to 30-50 sequences of intermediate size (on a typical modern laptop computer) due to memory requirements, but this can be circumvented using checkpoint algorithms, see [31].

## Conclusions

Previous corner-cutting methods define a compact part of the dynamic programming table for searching the best scored alignment. These methods become very inefficient when the number of sequences increases. We introduced a new progressive alignment method called Reticular Alignment, which obtains a set of optimal and suboptimal alignments at each step of the progressive alignment procedure. This set of alignments is represented by a network and are not directly embedded into the high-dimensional dynamic programming table. The set typically contains high-scored alignments that are usually not neighbours in the dynamic programming table (see, for example, the already mentioned Figure 2. in [22]). Therefore, the convex hull of the set of these alignments in the high dimensional dynamic programming table contains a Significantly larger set of alignments. Any previous corner-cutting method setting a convex part containing the set of alignments found by the Reticular Alignment method would need Significantly more memory and running time.

This novel corner-cutting approach allows the efficient search of the space of multiple sequence alignments for high-scored alignments. The method has a parameter which affects how much of the alignment space is explored. Furthermore, the Reticular Alignment method can be combined with any scoring scheme, and in this way, we were able to infer what is the importance of sophisticated scoring schemes and more exhaustive searches in finding accurate multiple sequence alignments.

The conclusion is that it is important to increase the search space for finding high-scored alignments. The Reticular Alignment method could find more accurate alignments than ClustalW even when the gap-scoring scheme was Significantly less sophisticated than the scoring scheme of ClustalW. For example, ClustalW gives different gap scores for hydrophilic and hydrophobic amino acids. This is considered to improve the alignment quality as hydrophobic amino acids are on the surface of globular proteins forming loops, and these loops undergo Significantly more insertion and deletion events than other parts of the proteins. Still, Reticular Alignment could generate more accurate alignments than ClustalW by merely extending the search space and without applying the above mentioned sophisticated scoring scheme of ClustalW. On the other hand, sophisticated scoring schemes are also necessary to get highly accurate multiple alignments. Combining sophisticated scoring schemes with the Reticular Alignment progressive alignment approach yielded a method whose accuracy is comparable to that of cutting-edge alignment methods. Without such sophisticated methods, the Reticular Alignment method only outperformed the ClustalX method, and were beaten by MAFFT and FSA in all accuracy measurements. Therefore it is also an important question how to find the scoring function that provides the most accurate multiple alignments. Kececioglu and Kim gave a fast linear programming-based method that finds parameter values that make given example alignments be optimal-scoring alignments of their strings [32]. Such extension of that approach for multiple sequence alignments would be desirable.

## Authors' contributions

IM proposed the extension of Waterman-Byers algorithm for aligning a network of alignments to a network of alignments, and implemented a prototype. AS developed the majority of the current RetAlign implementation. ÁN proposed the data structures and algorithms for efficient score calculation, and created the benchmarking framework to compare alignment programs. JH encouraged the discussions. All authors read and approved the final manuscript..

## References

[B1] Gusfield D Algorithms on Strings, Trees and Sequences: Computer Science and Computational Biology 1997 Cambridge University Press

[B2] Needleman SB Wunsch CD A general method applicable to the search for similarities in the amino acid sequence of two proteins J Mol Biol 1970 48 3 443 53 10.1016/0022-2836(70)90057-4 5420325

[B3] Sankoff D Cedergren RJ Time Warps, String Edits, and Macromolecules: The Theory and Practice of Sequence Comparison 1983 Addison-Wesley, Reading, Massachusetts 253 263 chap. Simultaneous comparison of three or more sequences related by a tree

[B4] Wang L Jiang T On the complexity of multiple sequence alignment J Comp Biol 1994 1 4 337 348 10.1089/cmb.1994.1.337 8790475

[B5] Fickett J Fast optimal alignment Nucleic Acids Research 1984 12 175 180 10.1093/nar/12.1Part1.175 6694900 PMC320994

[B6] Ukkonnen E Algorithms for approximate string matching Inform Control 1985 64 100 118 10.1016/S0019-9958(85)80046-2

[B7] Spouge J Fast optimal alignment CABIOS 1991 7 1 7 2004263 10.1093/bioinformatics/7.1.1

[B8] Hein J Wiuf C Knudsen B Moller MB Wibling G Statistical alignment: computational properties, homology testing and goodness-of-fit J Mol Biol 2000 302 265 279 10.1006/jmbi.2000.4061 10964574

[B9] Wu S Manber U Myers G Miller W An O(NP) sequence comparison algorithm Information Processing Letters 1990 35 6 317 323 10.1016/0020-0190(90)90035-V

[B10] Carrillo H Lipman D The multiple sequence alignment problem in biology SIAM Journal of Applied Mathematics 1988 48 1073 1082 10.1137/0148063

[B11] Lipman D Altschul S Kececioglu J A tool for multiple sequence alignment PNAS 1989 86 4412 4415 10.1073/pnas.86.12.4412 2734293 PMC287279

[B12] Gupta S Kececioglu J Schäffer A Improving the practical space and time efficiency of the shortest-paths approach to sum-of-pairs multiple sequence alignment J Comp Biol 1995 2 3 459 472 10.1089/cmb.1995.2.459 8521275

[B13] Hogeweg P Hesper B The alignment of sets of sequences and the construction of phyletic trees: An integrated method J Mol Evol 1984 20 2 175 186 10.1007/BF02257378 6433036

[B14] Feng DF Doolittle RF Progressive sequence alignment as a prerequisite to correct phylogenetic trees J Mol Evol 1987 25 351 360 10.1007/BF02603120 3118049

[B15] Higgins D Sharp P CLUSTAL: a package for performing multiple sequence alignment on a microcomputer Gene 1988 73 237 44 10.1016/0378-1119(88)90330-7 3243435

[B16] Thompson J Higgins D Gibson T ClustalW: improving the sensitivity of progressive multiple sequence alignment through sequence weighting, position-specific gap penalties and weight matrix choice Nucl Acids Res 1994 22 4673 4690 10.1093/nar/22.22.4673 7984417 PMC308517

[B17] Notredame C Higgins D Heringa J T-Coffee: A novel method for fast and accurate multiple sequence alignment J Mol Biol 2000 302 205 17 10.1006/jmbi.2000.4042 10964570

[B18] Katoh K Misawa K Kuma Ki Miyata T MAFFT: a novel method for rapid multiple sequence alignment based on fast Fourier transform Nucl Acids Res 2002 30 14 3059 3066 10.1093/nar/gkf436 12136088 PMC135756

[B19] Suchard MA Redelings BD BAli-Phy: Simultaneous Bayesian inference of alignment and phylogeny Bioinformatics 2006 22 16 2047 2048 10.1093/bioinformatics/btl175 16679334

[B20] Novák A Miklós I Lyngsø R Hein J StatAlign: An Extendable Software Package for Joint Bayesian Estimation of Alignments and Evolutionary Trees Bioinformatics 2008 24 20 2403 2404 10.1093/bioinformatics/btn457 18753153

[B21] Bradley R Roberts A Smoot M Juvekar S Do J Dewey C Holmes I Pachter L Fast Statistical Alignment PLoS Computational Biology 2009 5 e1000392 10.1371/journal.pcbi.1000392 19478997 PMC2684580

[B22] Zhu J Liu J Lawrence C Bayesian adaptive sequence alignment algorithms Bioinformatics 1998 14 25 39 10.1093/bioinformatics/14.1.25 9520499

[B23] Thompson J Koehl P Ripp R O P BAliBASE 3.0: latest developments of the multiple sequence alignment benchmark Proteins 2005 61 127 136 10.1002/prot.20527 16044462

[B24] Waterman MS Byers TH A dynamic programming algorithm to find all solutions in the neighborhood of the optimum Math Biosci 1985 77 179 188 10.1016/0025-5564(85)90096-3

[B25] Saitou N Nei M The neighbor-joining method: a new method for reconstructing phylogenetic trees Mol Biol Evol 1987 4 4 406 425 3447015 10.1093/oxfordjournals.molbev.a040454

[B26] Studier J Keppler K A note on the Neighbor-Joining algorithm of Saitou and Nei Mol Biol Evol 1988 5 6 729 731 3221794 10.1093/oxfordjournals.molbev.a040527

[B27] Hirschberg DS A linear space algorithm for computing maximal common subsequences Commun ACM 1975 18 6 341 343 10.1145/360825.360861

[B28] Ma B Wang Z Zhang K Alignment between Two Multiple Alignments Lecture Notes in Computer Science 2003 2676 254 265 full_text

[B29] Henikoff S Henikoff J Amino acid substitution matrices from protein blocks Proc Natl Acad Sci USA 1992 89 22 10915 10919 10.1073/pnas.89.22.10915 1438297 PMC50453

[B30] Durbin R Eddy S Krogh A Mitchison G Biological sequence analysis. Probabilistic models of proteins and nucleic acids 1998 Cambridge University Press

[B31] Tarnas C Hughey R Reduced space hidden Markov model training Bioinformatics 1998 14 401 406 10.1093/bioinformatics/14.5.401 9682053

[B32] Kececioglu J Kim E Simple and Fast Inverse Alignment Lecture Notes in Computer Science 2006 3909 441 455 full_text

